# Regulation of Osteoclastogenesis and Bone Resorption by miRNAs

**DOI:** 10.3389/fcell.2021.651161

**Published:** 2021-06-18

**Authors:** Kazuki Inoue, Courtney Ng, Yuhan Xia, Baohong Zhao

**Affiliations:** ^1^Arthritis and Tissue Degeneration Program, David Z. Rosensweig Genomics Research Center, Hospital for Special Surgery, New York, NY, United States; ^2^Department of Medicine, Weill Cornell Medicine, New York, NY, United States; ^3^Graduate Program in Cell and Developmental Biology, Weill Cornell Medicine Graduate School of Medical Sciences, New York, NY, United States

**Keywords:** osteoclast, miRNA, bone resorption, rheumatoid arthritis, osteoporosis

## Abstract

Osteoclasts are specialized bone-resorbing cells that contribute to physiological bone development and remodeling in bone metabolism throughout life. Abnormal production and activation of osteoclasts lead to excessive bone resorption in pathological conditions, such as in osteoporosis and in arthritic diseases with bone destruction. Recent epigenetic studies have shed novel insight into the dogma of the regulation of gene expression. microRNAs belong to a category of epigenetic regulators, which post-transcriptionally regulate and silence target gene expression, and thereby control a variety of biological events. In this review, we discuss miRNA biogenesis, the mechanisms utilized by miRNAs, several miRNAs that play important roles in osteoclast differentiation, function, survival and osteoblast-to-osteoclast communication, and their translational potential and challenges in bone biology and skeletal diseases.

## Introduction

Bone loss related to multiple diseases, such as rheumatoid arthritis (RA), osteoporosis, psoriatic arthritis and periodontitis, is a prevalent cause of disability in patients (Schett and Gravallese, 2012; Goldring et al., 2013). Bone destruction significantly diminishes the quality of life and simultaneously can increase the risk of mortality of these patients. Osteoclasts are giant, multinucleated cells that are derived from monocyte/macrophage lineage and specialize in bone resorption through proteolytic degradation and acid decalcification of the bone matrix. Osteoclast-mediated bone resorption is essential for skeletal development and normal bone remodeling. However, abnormal osteoclast formation and activation in pathological conditions play a crucial role in osteolysis. A broader understanding of the regulatory mechanisms that govern osteoclastogenesis will facilitate development of novel therapeutic strategies for patients subjected to pathological bone resorption.

Myeloid osteoclast precursors first undergo differentiation into mononuclear osteoclasts in response to the master osteoclastogenic cytokine: receptor activator of nuclear factor-κB ligand (RANKL) accompanied by macrophage colony-stimulating factor (M-CSF) and immunoreceptor tyrosine-based activation motif (ITAM)-mediated co-stimulatory signaling pathways. Mononuclear osteoclast precursors then fuse into giant, mature polykaryons driven by the expression of cell-cell fusion genes, such as DC-Stamp, ATP6v0d2 and Gα13 (Yagi et al., 2005; Lee et al., 2006; Nakano et al., 2019). Osteoclastogenesis is delicately regulated and influenced in order to maintain the balance of osteoclastogenic and anti-osteoclastogenic mechanisms (Zhao and Ivashkiv, 2011). RANKL induces a broad range of signaling cascades including canonical and non-canonical NF-kB pathways, mitogen-activated kinase (MAPK) pathways and calcium signaling. These pathways activate downstream transcription factors to drive osteoclast differentiation, such as c-Fos, nuclear factor of activated T cells c1 (NFATc1), and B lymphocyte-induced maturation protein-1 (Blimp1). On the other hand, negative regulators mediating intrinsic anti-osteoclastogenic mechanisms, such as differentially expressed in FDCP 6 homolog (Def6), interferon regulatory factor (IRF8) and v-maf musculoaponeurotic fibrosarcoma oncogene family protein B (MafB), function as brakes to restrain excessive osteoclast formation and bone resorption (Humphrey et al., 2005; Asagiri and Takayanagi, 2007; Zhao B. et al., 2009; Nakashima and Takayanagi, 2011; Zhao and Ivashkiv, 2011; Xu and Teitelbaum, 2013; Li et al., 2014; Boyce et al., 2015; Binder et al., 2017). Some regulators were identified to preferentially limit pathological osteoclastic bone resorption, such as RBP-J and NF-κB p100 (Yao et al., 2009; Zhao et al., 2012). These regulators and associated mechanisms interact to form regulatory networks that coordinately modulate osteoclastic gene expression to govern osteoclast differentiation and function.

Over the past decade, epigenetic studies have gained rapidly growing research and clinical attention. Epigenetics investigates heritable changes in gene expression and cellular phenotype that do not involve alterations in DNA sequence, which provides a new level and dimension of understanding of gene expression. Major epigenetic mechanisms include DNA methylation, chromatin remodeling, histone posttranslational modifications and non-coding RNAs (ncRNAs) (Zhang and Cao, 2019). These mechanisms often act in concert to play crucial roles in the regulation of gene expression extensively involved in diverse biological and disease settings, such as cell proliferation and differentiation, development, cardiovascular disease, diabetes, autoimmune diseases, and cancer. In human genome, ncRNAs constitute more than 90% of the genome transcripts (Hangauer et al., 2013). ncRNAs consists of multiple classes of RNA that are not translated into proteins, which are classified into two categories by size; long non-coding RNAs (lncRNAs) (>200 nt) and small RNAs (<200 nt). Small RNAs include microRNAs (miRNAs), short interfering RNAs (siRNAs) and Piwi-interacting RNAs (piRNAs) (Kim et al., 2009; Esteller, 2011). Recently, a unique class of ncRNAs transcribed from sequences of enhancer regions, called enhancer RNAs (eRNAs), have been identified (Kim et al., 2010; Lam et al., 2014). Genome-wide cap analysis of gene expression (CAGE) identified eRNA regions at the Nrp2, Dcstamp, and Nfatc1 gene loci in osteoclasts. These eRNAs are necessary for the transcription of these genes that are important for osteoclast differentiation (Sakaguchi et al., 2018). To date, functions of lncRNAs in osteoclastogenesis are largely unknown. The most well-characterized ncRNAs in myeloid cells are miRNAs. In this review, we discuss miRNA biogenesis, several miRNAs that play important roles in osteoclast differentiation, function, survival, osteoblast-to-osteoclast communication, the mechanisms controlled by miRNAs, and their translational potential and challenges in bone biology and skeletal diseases (Table 1).

**TABLE 1:** Osteoclastogenesis and bone metabolism mediated by miRNAs.

**microRNAs**	**Targets**	**miRNA-expressing Cells**	***In vitro* effects on osteoclasts**	***In vivo* effects (KO,Tg mice, inhibitor, agonist)**	**Clinical significance**
miR-21	PDCD4 (Hu et al., 2017) FasL (Sugatani and Hruska, 2013)	Mouse BMMs	Osteoclastogenic promotion (Sugatani and Hruska, 2013; Hu et al., 2017)	miR-21 KO mice: decreased bone resorption and osteoclastogenesis (Hu et al., 2017)	miR-21 expression is increased in osteoporotic human serum and inversely correlated with BMD (Sugatani and Hruska, 2013)
miR-31	RhoA (Mizoguchi et al., 2013)	Mouse BMMs	Osteoclastogenic promotion (Mizoguchi et al., 2013)	NA	NA
miR-214	PTEN (Zhao et al., 2015) TRAF3 (Liu et al., 2017)	RAW264.7 cells Mouse BMMs	Osteoclastogenic promotion (Zhao et al., 2015)	miR-214 transgenic mice: reduced bone mass, increased osteoclastogenesis (Zhao et al., 2015) miR-214 KO mice: the development of osteolytic breast cancer metastasis is prevented (Liu et al., 2017)	miR-214-3p is upregulated in osteolytic bone metastasis of breast cancer (Liu et al., 2017)
miR-182	PKR (Inoue et al., 2018) Foxo3,Maml1 (Miller et al., 2016)	Mouse BMMs Human CD14^+^ monocytes	Osteoclastogenic promotion (Miller et al., 2016; Inoue et al., 2018)	miR-182 KO mice: increased bone mass, decreased bone resorption and osteoclastogenesis (Inoue et al., 2018) miR-182 transgenic mice: decreased bone mass, increased bone resorption and osteoclastogenesis (Inoue et al., 2018) miR-182 inhibitor: bone loss in OVX and inflammatory arthritis is prevented (Inoue et al., 2018)	miR-182 expression is higher in RA patients, and TNF blockade therapy (TNFi) with Enbrel suppresses miR-182 expression levels in RA (Inoue et al., 2018)
miR-183	HO-1 (Ke et al., 2015)	Mouse BMMs	Osteoclastogenic promotion (Ke et al., 2015)	NA	NA
miR-7b	DC-STAMP (Dou et al., 2014)	RAW264.7 cells	Osteoclastogenic inhibition (Dou et al., 2014)	NA	NA
miR-124	Rab27a (Tang et al., 2017)	Mouse BMMs	Osteoclastogenic inhibition (Lee et al., 2013; Tang et al., 2017)	Pre-miR-124 suppresses bone destruction and osteoclast in AIA rats (Nakamachi et al., 2016)	NA
miR-141	Calcr (Ell et al., 2013) EphA2 (Yang S. et al., 2018)	Mouse BMMs	Osteoclastogenic inhibition (Ell et al., 2013; Yang S. et al., 2018)	miR-141 mimic suppresses osteolytic bone metastasis (Ell et al., 2013) and osteoporosis (Yang S. et al., 2018)	miR-141 expression is decreased in osteoporosis patients (Yang S. et al., 2018)
miR-503	RANK (Chen et al., 2014)	Human CD14^+^ monocytes	Osteoclastogenic inhibition (Chen et al., 2014)	miR-503 mimic suppresses bone resorption and osteoclastogenesis in OVX model (Chen et al., 2014)	miR-503 is downregulated in CD14^+^ PBMCs of postmenopausal osteoporotic patients (Chen et al., 2014)
miR-125b	Prdm1 (Minamizaki et al., 2020)	Osteoblasts	Osteoclastogenic inhibition (Minamizaki et al., 2020)	miR-125 trangenic mice: reduced bone resorption and osteoclastogenesis in OVX model, LPS-induced calvarial bone loss model, and sciatic neurectomy (NX)-induced osteoporosis mouse model (Minamizaki et al., 2020)	NA
miR-155	MITF, SOCS1 (Zhang et al., 2012) TAB2 (Sul et al., 2018)	Mouse BMMs	Dual effects (Zhang et al., 2012; Zhao et al., 2017; Sul et al., 2018)	NA	NA
miR-223	NFI-A (Sugatani and Hruska, 2009; Xie et al., 2015)	RAW264.7 cells	Dual effects (Sugatani and Hruska, 2007, 2009; Kagiya and Nakamura, 2013; Xie et al., 2015)	NA	NA
					

### Post-transcriptional Regulation of Target Gene Expression by miRNAs

**FIGURE 1:**
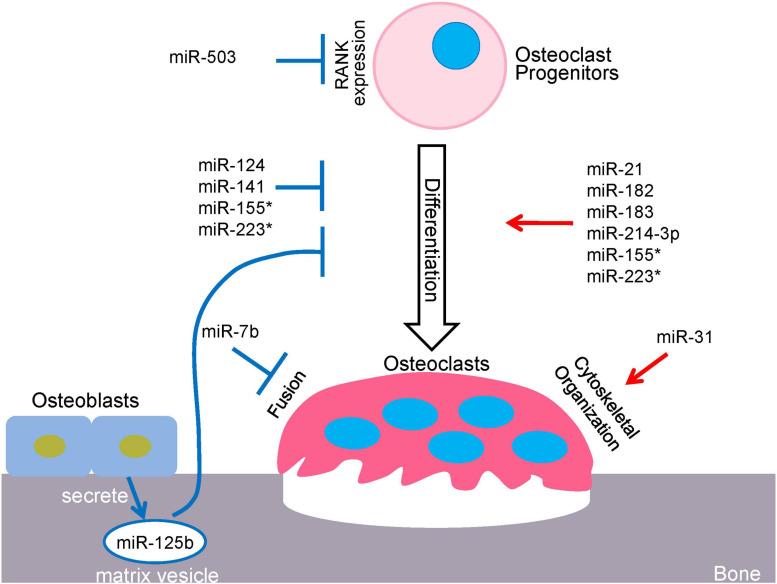
miRNA-mediated regulatory network in osteoclastogenesis. *miR-155 and miR223 play dual roles in osteoclastogenesis.

MicroRNAs (miRNAs) constitute a class of small single-stranded non-coding RNAs (about 22 nucleotides), that function in diverse regulatory pathways through gene repression of their target mRNAs via post-transcriptional mechanisms (Lagos-Quintana et al., 2001; Bartel, 2004). miRNAs commonly bind to multiple target sequences at the 3′ untranslated region (3′-UTR) of mRNAs, through perfect complementary pairing of nucleotides 2–7 at the 5′ end of the miRNA (the miRNA seed region) (Bartel, 2009). These molecules repress gene expression via mRNA degradation or translational inhibition of their target mRNAs, or a combination of these mechanisms (Ling et al., 2013). A series of processes is required to generate mature miRNAs, beginning with the transcription of long precursor RNAs, called primary miRNAs (pri-miRNAs), by RNA polymerase II (Lee et al., 2004). In the nucleus, long pri-miRNAs are cropped into hairpin structures by the microprocessor complex, which consists of the RNase III enzyme, Drosha and RNA binding protein, DiGeorge syndrome critical region 8 (DGCR8). This process produces the precursor miRNAs (pre-miRNAs) that are approximately 60–70 nucleotides (Lee et al., 2003; Gregory et al., 2004). Pre-miRNAs are then exported from the nucleus by Exportin-5 (Exp5) into the cytoplasm where they are further cleaved by the RNase III enzyme, Dicer (Lund et al., 2004). Upon recognition, Dicer cleaves the pre-miRNA at the double-stranded RNA stem and the terminal loop to generate a mature ∼22 nucleotide miRNA duplex (Park et al., 2011). The miRNA duplex consists of two strands that are often referred to as a guide and a passenger strand. The passenger strand is typically degraded while the guide strand of the mature miRNA is incorporated into the RNA-induced silencing complex (RISC), which comprises Dicer, transactivating response RNA-binding protein (TRBP) and Argonaute proteins (AGO1-4) (Chendrimada et al., 2005; Peters and Meister, 2007). Active RISC is guided by miRNA to complementary target mRNAs and downregulates gene expressing by inducing mRNA degradation, translational repression, or a combination of the two mechanisms. AGO2 was identified to have catalytic slicer activity that can facilitate degradation of specific target mRNA. The target mRNAs sharing the same recognition sequences could compete for miRNA binding and influence the expression of each target gene. A single miRNA can recognize and regulate multiple mRNA targets, and consequently, may have a wide range and substantial effect on gene expression networks. Additionally, within the same cell types, the targets of an miRNA can be variable in response to differential environmental settings. Accordingly, miRNA-targeted gene regulation is both specific and sensitive to environmental changes. Multiple miRNAs can also be involved in the same biological processes, as exemplified by the miRNAs mentioned in this review, which play crucial and exclusive roles in osteoclast differentiation and function (Figure 1).

### miRNAs That Positively Regulate Osteoclastogenesis

#### miR-21

In mammalian cells, miR-21 is most widely recognized as an oncogenic miRNA and its upregulation is associated to the progression of various cancers (Feng and Tsao, 2016). In addition, miR-21 is induced by inflammatory cytokines and plays regulatory roles in various cells. For example, miR-21 is induced by tumor necrosis factor-α (TNF-α) in endothelial cells (Yang D. et al., 2018), by interleukin-1β (IL-1β) in chondrocytes (Ma et al., 2020) and by interleukin-6 (IL-6) in myeloma cells (Loffler et al., 2007). Recently, miR-21 has gained recognition within the processes of bone metabolism and disease. *In vitro* studies have shown that RANKL induces miR-21 expression and promotes osteoclastogenesis via down-regulation of programmed cell death 4 (PDCD4), which subsequently regulates the c-Fos/NFATc1 axis (Sugatani et al., 2011). The pro-osteoclastic function of miR-21 is further demonstrated *in vivo* using miR-21 knockout mice, in which miR-21 deficiency inhibits bone resorption and osteoclastogenesis via targeting PDCD4 (Hu et al., 2017). miR-21 is found to be downregulated by bisphosphonate in lung cancer patients with osteolytic bone metastasis (Zhao et al., 2020). Another study revealed that estrogen suppresses miR-21 biogenesis, leading to upregulation of Fas Ligand (FasL), which is a target of miR-21 in osteoclasts and induces osteoclast cell death in an autocrine manner (Sugatani and Hruska, 2013). Thus, miR-21 also contributes to osteoclast survival and estrogen-mediated osteoclastogenesis.

#### miR-31

miR-31 is induced by RANKL, and positively regulates cytoskeletal organization during osteoclastogenesis and bone resorption activity. It does so by targeting expression of RhoA to optimize actin ring formation in osteoclasts, which is crucial for both cytoskeletal organization and bone resorption (Mizoguchi et al., 2013). In addition, inflammatory cytokines can regulate miR-31 expression. For instance, miR-31 is induced by TNF-α in cord blood mononuclear cells (Kim et al., 2020), by IL-1β in HUVEC endothelial cells (Zhong et al., 2017) and by IL-6 in HuCC-T1 cholangiocarcinoma cell line (Ishigami et al., 2018).

#### miR-214

The crosstalk and intercellular interaction between osteoclasts and osteoblasts are necessary for maintaining bone homeostasis and regulating bone remodeling. This communication involves secretory factors or cell-cell contact signaling, such as via RANKL, OPG, TGF-β1, IGF-1, Sema4D, Sema3A, ephrinB2/EphB4, Cthrc1, and Wnt16 (Zhao et al., 2006; Cao, 2011; Negishi-Koga et al., 2011; Hayashi et al., 2012; Takeshita et al., 2013; Moverare-Skrtic et al., 2014), and can be facilitated by bone-derived exosomes (Xie et al., 2017). Extracellular vesicles, or exosomes, are essential in coordinating intercellular communication through delivery of proteins, lipids, and nucleic acids including DNA, mRNA and miRNA. Moreover, tumor-derived exosomes are crucial in promoting tumor microenvironment and metastasis (Becker et al., 2016). Recent evidence has identified a role of osteoclast-derived exosomal miR-214-3p in mediating inhibition of osteoclast-directed osteoblast formation (Li et al., 2016). Osteoclastic miR-214-3p was found in association with reduced bone formation, and elevated levels of miR-214-3p were found in whole serum, serum exosome and bone tissue from elderly women with fractures and OVX mice. Osteoclast-specific miR-214-3p overexpression mice (OC-miR-214-3p) exhibited elevated exosomal miR-214-3p in serum and reduced bone formation, and this was rescued by injection of antagomiR-214-3p delivered by osteoclast-targeted (D-Asp8)-liposome (Liang et al., 2015). *In vitro* evidence revealed that osteoclastic exosomal miR-214-3p can transfer to osteoblasts and inhibit osteoblast activity. Consistently, *in vivo* osteoclast-targeted inhibition of miR-214-3p was able to enhance bone formation in aging OVX mice. These results highlight the impact of exosomal miR-214-3p as a mediator for bone cell communication on bone disease and related crosstalk. Wang et al. also provided evidence supporting the inhibitory role of miR-214 on bone formation and osteoblast activity via direct targeting of ATF4, an essential transcription factor for osteoblastogenesis and function (Yang et al., 2004; Wang et al., 2013; Li et al., 2016).

Moreover, miR-214-3p was found to be upregulated during osteoclastogenesis and positively regulates the differentiation process through activating PI3K/Akt/NFATc1 pathway by directly targeting phosphatase and tensin homolog (PTEN) (Zhao et al., 2015). Inhibition of miR-214-3p attenuated, while overexpression of miR-214-3p promoted, *in vitro* osteoclastogenesis. OC-miR-214-3p transgenic mice exhibited reduced PTEN expression levels, enhanced osteoclast activity and reduced bone mass, proposing potential as a therapeutic target for osteoporosis. Previous studies have also shown that PTEN is a target of miR-214-3p, which promotes T cell activation and proliferation (Jindra et al., 2010). Upregulation of miR-214-3p was also observed in osteolytic bone metastasis of breast cancer, which was consistently associated with elevated osteoclast activity in nude mice xenografted with human breast cancer cells (Liu et al., 2017). Breast cancer cells are able to induce miR-214-3p expression and interestingly, genetic ablation of osteoclast-specific miR-214-3p prevented the development of osteolytic breast cancer metastasis. This study also revealed that mechanistically, miR-214-3p promotes bone resorption through direct targeting of TNF-α Receptor Associated Factor 3 (TRAF3), and osteoclast-targeted treatment of Traf3 3′UTR plasmid attenuated excessive bone resorption in OC-miR-214-3p mice. In addition, miR-214 is decreased by TNF-α in HT29 colorectal adenocarcinoma cells (Li et al., 2017), induced by IL-1β in primary chondrocytes (Xu et al., 2021) and induced by IL-6 in human colonocytes (Polytarchou et al., 2015). This regulation of miR-214 expression by inflammatory cytokines suggests potential roles for miR-214 in inflammatory bone metabolism. Collectively, these studies strongly support the potential of miR-214-3p as a therapeutic target through regulating both osteoblasts and osteoclasts to treat bone disorders such as osteoporosis and cancer bone metastasis.

#### miR-182/miR-183

The miR-183 cluster, an miR family comprised of miR-182, miR-183 and miR-96, is well recognized to be functionally involved in development and also highly expressed and implicated in cancers, neurological disorders, autoimmune diseases (Dambal et al., 2015), and more recently, innate immunity (Singaravelu et al., 2019). miR96 was not detected during osteoclastogenesis (Miller et al., 2016), while miR-183 expression was found to be elevated by RANKL and promoted *in vitro* RANKL-induced osteoclastogenesis via targeting of heme oxygenase-1 (HO-1) (Ke et al., 2015). Recent studies revealed the functions of miR-182 in cell cycle, cell growth, cancer progression, T lymphocyte expansion and Th17 function (Stittrich et al., 2010; Ichiyama et al., 2016; Yao et al., 2019). miR-182 and miR-183 are induced by IL-6 but not IL-1β in Th17 cells and contribute to Th17 cell pathogenicity (Ichiyama et al., 2016).

Through genome-wide miRNA expression profiling via high-throughput miRNA-sequencing, our group identified miR-182 as a TNF-α-induced miRNA in mouse BMMs during inflammatory osteoclastogenesis (Miller et al., 2016). We further elucidated the role of miR-182 in regulating bone metabolism in both physiological and pathological conditions, such as those which occur in osteoporosis and rheumatoid arthritis (RA) (Inoue et al., 2018). Postmenopausal osteoporosis and RA are characterized by pathological bone destruction resulting from osteolysis conducted by osteoclasts. miRNA-based therapeutics for these diseases appear promising, though underdeveloped. Through a series of subsequent studies, we identified miR-182 as a key osteoclastogenic regulator, provided evidence revealing a novel regulatory network mediated by miR-182 in osteoclastogenesis, imparted therapeutic implications of targeting miR-182 to promote osteoprotection, and highlighted significant correlation between miR-182 and RA.

Our findings on miR-182 reveal great significance to the understanding of its role in bone metabolism, and potential impact on therapeutics and drug development for the treatment of bone diseases. Utilizing complementary genetic approaches (myeloid-specific miR-182 KO and Tg mice), miR-182 was identified as a key positive regulator of osteoclastogenesis. OVX-induced osteoporosis, which serves as a model for postmenopausal osteoporosis, and inflammatory arthritis, which mimics RA, were the disease models applied in this study for investigating translational significance. Genetic ablation or pharmacological inhibition of miR-182 prevented pathological bone loss in both models, suggesting a key role for miR-182 in bone protection. To test the efficacy of therapeutically targeting miR-182, chitosan (CH) nanoparticles were utilized to effectively and safely deliver miR-182 inhibitors to bone marrow and specifically target osteoclast lineage. The treatment with miR-182 inhibitors dramatically suppressed excessive osteoclast formation and bone resorption in these models without immune suppression, thus providing proof-of-principle that miR-182 inhibition presents significant clinical value for treating bone destruction. We also revealed and elucidated the miR-182-PKR-IFN-β axis through the identification of protein kinase double-stranded RNA-dependent (PKR), which is a crucial target of miR-182, and a novel inhibitor of osteoclastogenesis. PKR attenuates osteoclastogenesis by upregulating the endogenous IFN-β-mediated autocrine feedback loop. For the first time, a miR-182-PKR axis is revealed to be responsible for suppressing this IFN-β signaling induced by RANKL to activate osteoclastogenesis, and this network is involved in fine tuning the osteoclastogenic program. In the context of disease, the miR-182-PKR-IFN-β axis exhibits aberrant expression level changes including elevation of miR-182, and downregulation of PKR and IFN-β levels in the PBMCs from RA patients in comparison to healthy donors. Given the role of TNF-α in the pathogenesis of RA, TNF-α blockade therapy (TNFi) with Enbrel suppressed miR-182 expression levels and elevated PKR and IFN-β expression levels, and decreased osteoclastogenesis in RA patients-derived PBMCs. The osteoclastogenic capacity of PBMCs from RA patients is significantly correlated with the expression pattern of the miR-182-PKR-IFN-β axis. Both murine and human data provide strong evidence supporting the role of miR-182 in osteoclastogenesis, and translational promise of targeting miR-182 to treat pathological bone destruction.

### miRNAs That Negatively Regulate Osteoclastogenesis

#### miR-7b

miR-7b has been reported to participate in the regulation of B cell receptor (BCR) signaling, which is enhanced in autoreactive B cells from systemic lupus erythematosus (SLE) (Wu et al., 2014). B cell hyper-responsiveness in SLE results from the downregulation of Phosphatase and tensin homolog (PTEN) by upregulated miR-7 (Wu et al., 2014). miR-7b is induced by a cocktail of IL-1β, IFN-γ, and TNF-α in the MIN6 insulinoma cell line (Bravo-Egana et al., 2012), suggesting its involvement in inflammation.

Within the process of osteoclastogenesis, maturation of multi-nucleated osteoclasts requires cell-cell fusion of mononuclear osteoclast precursors. Bone disease, such as osteoporosis can result from deregulated osteoclastic bone-resorbing activity when osteoclast multinucleation is defected. The role of miR-7b was revealed through its direct targeting of dendritic cell-specific transmembrane protein (DC-STAMP), which is a critical mediator of the osteoclast fusion process (Yagi et al., 2005; Dou et al., 2014). Positive regulation of DC-STAMP expression is mediated by NFATc1, c-Fos, and strawberry notch homolog 2 (Sbno2) (Maruyama et al., 2013). At the post-transcriptional level, miR-7b negatively regulates DC-STAMP, resulting in the inhibition of osteoclast fusion. Overexpression of miR-7b represses the expression of other fusogenic genes such as CD47, ATP6v0d2 and OC-STAMP, as well as osteoclast specific genes such as Nfatc1 and OSCAR via DC-STAMP inhibition. These studies characterize the crucial role of miR-7b in osteoclast multinucleation and function.

#### miR-124

miR-124 is, notably, abundantly expressed in neuronal cells, promoting cell differentiation (Lagos-Quintana et al., 2002; Makeyev et al., 2007), and also known as a critical regulator of immune function and implicated in inflammatory disorders (Qin et al., 2016). Expression of miR-124 is inversely correlated with activation of microglia during inflammation such as experimental autoimmune encephalomyelitis (EAE) (Ponomarev et al., 2011). Overexpression of miR-124 was found to promote microglia quiescence through directly targeting C/EBP-α and its downstream target, PU.1. This deactivation of macrophages suppresses CNS inflammation and inhibits EAE. In addition, inflammatory cytokines regulate the expression of miR-124. For example, TNF-α in keratinocytes (Yang et al., 2017) or IL-1β in lumbar (Willemen et al., 2012) was reported to decrease miR-124.

In addition to its effect on macrophage phenotypes in the CNS, miR-124 was shown to inhibit osteoclastogenesis in mouse BMMs by suppressing the expression of NFATc1 (Lee et al., 2013). miR-124 is downregulated in BMMs and osteoclasts from OVX mice, resulting in excessive bone resorption and destruction (Tang et al., 2017). This study also revealed a functional target of miR-124, Rab27a, which regulates multinucleation and transport of lysosome-related organelles (Shimada-Sugawara et al., 2015). Furthermore, miR-124 was reported to be downregulated in the ankles of adjuvant-induced arthritis (AIA) rats (Nakamachi et al., 2016). Administration of pre-miR-124 via injection into the ankles of AIA rats strongly suppressed bone destruction and reduced osteoclastogenesis via direct targeting of NFATc1. These findings support the novel role of miR-124 as a suppressor of osteoclastogenesis.

#### miR-141

miR-141 is identified as a tumor inhibitor, as well as a significant regulator participating in tumor-induced osteolytic bone metastasis. Conditioned medium (CM) from bone metastatic cell lines such as 4T1.2 and TSU-Pr-B2 cells were shown to be sufficient in inducing osteoclastogenesis, and miRNA microarray analysis revealed significant downregulation of miR-141 in osteoclasts under the treatment (Ell et al., 2013). Overexpression of miR-141 suppressed osteoclast differentiation via targeting of MITF and calcitonin receptor (CALCR), with observed downregulation of osteoclast marker expression such as NFATc1, CtsK, and PU.1. Systemic treatment of miR-141 was able to inhibit osteoclastogenesis and activity *in vivo*, as well as suppress osteolytic activity of breast cancer bone metastasis *in vivo*, accompanied with reduced osteoclast differentiation and activity. Further support of the inhibitory role of miR-141 on osteoclastogenesis and bone resorbing activity was established through the study of aged osteoporotic rhesus monkeys conducted by Yang S. et al. (2018). Both osteoporotic patients and aged rhesus monkeys exhibited downregulated expression of miR-141 in bone tissues. Targeted delivery of miR-141 to osteoclasts using Asp (Aspartic acid) 8-PU (polyurethane) nanoparticles inhibited bone loss, osteoclast differentiation and bone resorption activity *in vivo*, as well as in *in vitro* culture of rhesus monkey osteoclasts. They also demonstrated that miR-141 functionally targets Calcr and ephrin type-A receptor 2 precursor (EphA2) to inhibit osteoclastogenesis. Collectively, these data present the critical role of miR-141 in suppression of osteoclastogenesis and bone resorption, implicated in aberrant bone loss in bone disease and cancer.

#### miR-503

miR-503 has been identified in numerous cancer types via miRNA expression profiling analyses, including human retinoblastoma, glioblastoma, hepatocellular carcinomaand non-small cell lung cancer (NSLC), many of which support a tumor-suppressive role of miR-503 (Zhao J.J. et al., 2009; Zhou and Wang, 2011; Yang et al., 2014; Zhang et al., 2014). It has also been demonstrated that miR-503 can induce G1 cell cycle arrest to promote myogenic differentiation and similarly through promoting cell cycle quiescence, inhibit progression of osteosarcoma and colon cancer cell proliferation (Sarkar et al., 2010; Chong et al., 2014; Chang et al., 2015). In contrast, miR-503 expression has been reported to be upregulated in certain cancers and identified as a tumor-associated miRNA that directly targets Bcl2 apoptosis regulator to promote apoptosis of dendritic cells, indicating a mechanism for tumor immunotolerance (Min et al., 2013).

In the context of bone disorders, miR-503 was identified to be dramatically downregulated in CD14^+^ PBMCs of postmenopausal osteoporotic patients in comparison to postmenopausal healthy women. Following discontinuation of denosumab (an anti-RANKL antibody) treatment, miR-503 expression is decreased in patients with vertebral fractures (Anastasilakis et al., 2017). These findings suggest a crucial role for miR-503 in bone metabolism (Chen et al., 2014). Overexpression and silencing of miR-503 in CD14^+^ PBMCs confirmed its inhibitory role on osteoclastogenesis and its direct targeting of RANK, the receptor for RANKL. Treatment with agomiR-503 protected OVX mice from bone loss by inhibiting bone resorption and increased bone mass. Consistently, estrogen upregulated miR-503 expression *in vitro* and *in vivo*, confirming its contribution to the pathogenesis of postmenopausal osteoporosis and potential as a therapeutic target.

#### miR-125b

Bone matrix growth factors, such as TGF-β and IGF-1, play essential roles in coupling bone resorption and formation (Tang et al., 2009; Xian et al., 2012). Recently, miR-125b has been identified as a bone matrix-embedded coupling factor that is produced by osteoblasts (Minamizaki et al., 2020). miR-125b is enveloped by matrix vesicles (MVs) and accumulates in bone matrix. The osteoblast-derived MVs containing miR-125b are incorporated into osteoclast precursors and inhibit osteoclastogenesis. Mice overexpressing miR-125b in osteoblasts using human osteocalcin promoter show high bone mass phenotype and reduced number of bone-resorbing osteoclasts, without affecting osteoblasts and bone formation. miR-125b directly targets Prdm1, a key transcriptional factor of osteoclastogenesis, in osteoclast precursors. Overexpression of miR-125b in osteoblasts abrogates bone loss in OVX-induced osteoporosis mouse model, LPS-induced calvarial bone loss model, and sciatic neurectomy (NX)-induced osteoporosis mouse model. The MV containing miR-125b is a novel coupling factor of osteoblast-osteoclast communication. MVs containing miR-125b might be potential therapeutic targets for pathological bone loss.

### miRNAs That Play Dual Roles in Osteoclastogenesis

#### miR-155

miR-155 plays crucial roles in cell development and function of various immune cells involved in both innate and adaptive immunity and is implicated in pathological conditions such as cancer (O’Connell et al., 2007, 2009; Thai et al., 2007; Vigorito et al., 2007; Teng et al., 2008). It has been shown that inflammatory cytokines and stimuli, such as TNF-α and lipopolysaccharide (LPS), can activate miR-155 expression, which in turn regulates proliferation, differentiation and function of macrophages and dendritic cells (O’Connell et al., 2007, 2009; Ceppi et al., 2009; Lind et al., 2015).

In addition to its role in immune cells, miR-155 has been revealed to inhibit RANKL-induced osteoclastogenesis through TGF-β1/Smad4 signaling and by mediating the suppressive effect of IFN-β on osteoclast differentiation through targeting microphthalmia-associated transcription factor (MITF), suppressor of cytokine signaling 1 (SOCS1), and PU.1 (Zhang et al., 2012; Zhao et al., 2017). However, in the context of LPS-mediated inflammatory bone loss, it has been recently reported that miR-155 is induced by LPS and enhances autophagy to increase osteoclast differentiation and activity through targeting transforming growth factor β-activated kinase 1-binding protein 2 (TAB2) (Sul et al., 2018). Taken together, miR-155 plays an inhibitory role in RANKL-induced osteoclastogenesis but enhances LPS-mediated inflammatory osteoclastogenesis.

#### miR-223

miR-223 was known to be preferentially expressed in hematopoietic cells and identified as a key regulator of myeloid cell differentiation and activation, particularly neutrophils and macrophages, and therefore, a regulator of innate immune response through myeloid cell homeostasis (Fukao et al., 2007; Yuan et al., 2018). Studies have demonstrated that miR-223 has the abilities to promote macrophage polarization to adopt anti-inflammatory phenotype and inhibit activation of macrophages and subsequent inflammatory response through targeting signal transducer and activator of transcription 3 (STAT3) (Chen et al., 2012) and inflammasome sensor-NLRP3 (Bauernfeind et al., 2012). In contrast, miR-223 expression is downregulated under inflammatory conditions activated by Toll-like receptor (TLR) ligands, such as LPS in macrophages (Chen et al., 2012; Zhang et al., 2017). However, elevated expression of miR-223 in CD68^+^ macrophages, CD14^+^ monocytes, and CD4^+^ T cells from synovium of rheumatoid arthritis (RA) patients was reported (Li et al., 2012; Shibuya et al., 2013). miR-223 expression is also induced by TNF-α in MH7A fibroblast-like synoviocyte cell line (Moriya et al., 2017). In osteoclast precursor cells, overexpression of miR-223 was shown to downregulate osteoclastogenesis (Sugatani and Hruska, 2007; Kagiya and Nakamura, 2013). However, conflicting studies have reported that in osteoclast precursor cells, a positive feedback loop exists whereby M-CSF induces transcription factor, PU.1, which stimulates miR-223 expression, resulting in downregulation of its target, nuclear factor I A (NFIA). The downregulation of NFIA expression allows for upregulation of M-CSF receptor levels and consequently, promotion of osteoclastogenesis (Sugatani and Hruska, 2009; Xie et al., 2015). PU.1, c-Fos, MITF, and NFATc1 levels are upregulated by M-CSF and RANKL during osteoclastogenesis. These results suggest that miR-223 is essential for osteoclast differentiation and function. However, the mechanisms underlying the dual effects of miR-223 on osteoclastogenesis are not well-understood.

## Closing Remark

miRNAs are involved in numerous molecular pathways and cellular events through targeting specific genes. Recent studies have identified competing endogenous RNAs (ceRNAs), which comprise of mRNAs, pseudogenes, long non-coding RNAs (lncRNAs) and circular RNAs (circRNA) (Cesana et al., 2011; Salmena et al., 2011; Tay et al., 2014). These ceRNAs communicate with each other to compete for shared miRNAs, acting as natural miRNA “sponges,” to block miRNA binding and repression of its targets. ceRNAs impose a regulatory mechanism upon miRNAs, adding another level of complexity to miRNA-mediated gene regulation. The mechanisms of miRNA-ceRNA networks is another channel to unpack in further exploration and understanding of gene regulation by different RNA species in specific processes, pathways and tissues, which are especially important for understanding their translational implication.

Targeting miRNAs has shown promising therapeutic potential in a few clinical trials, such as in the treatment of diabetes, cancer and hepatitis C (Janssen et al., 2013; Chakraborty et al., 2017; Rupaimoole and Slack, 2017). These inspiring scientific advances highlight the clinical significance of the emerging field of miRNA and miRNA-based therapeutics. The miRNAs discussed in this review strongly support the potential of therapeutic targeting of miRNAs to prevent and treat pathological bone loss, such as that occurring in RA, osteoporosis and bone cancer metastasis. However, further work is needed in elucidating miRNA-mediated gene regulation networks specific to these diseases in order to successfully enhance the development of therapeutic approaches. In addition, generating an ideal miRNA-based drug delivery method presents challenges, such as ensuring stability, specificity and efficacy of targeting, and minimization of toxicities and off-target effects. Future studies and technological developments are expected to address these challenges in the clinical development of miRNA-based drugs.

## Author Contributions

BZ designed, wrote, and edited the manuscript. KI wrote and edited the manuscript. CN and YX helped to writing and editing the manuscript. All authors contributed to the article and approved the submitted version.

## Conflict of Interest

The authors declare that the research was conducted in the absence of any commercial or financial relationships that could be construed as a potential conflict of interest.
